# Decoration of Cotton Fibers with a Water-Stable Metal–Organic Framework (UiO-66) for the Decomposition and Enhanced Adsorption of Micropollutants in Water

**DOI:** 10.3390/bioengineering5010014

**Published:** 2018-02-12

**Authors:** Marion Schelling, Manuela Kim, Eugenio Otal, Juan Hinestroza

**Affiliations:** 1 Department of Fiber Science, Cornell University, Ithaca, NY 14853, USA; ms2567@cornell.edu; 2 Division of Porous Materials, UNIDEF, CITEDEF, CONICET, S. J. B de la Salle 4397, Villa Martelli (B1603ALO), Buenos Aires 1603, Argentina; mlkrei@gmail.com (M.K.); Eugenioh@gmail.com (E.O.); 3 Laboratory for Materials Science and Technology, FRSC-UTN, Av. Inmigrantes 555, Río Gallegos 9400, Argentina

**Keywords:** UiO-66, MOF, cotton fabric, functionalized cellulose, methylchlorophenoxypropionic acid, paracetamol

## Abstract

We report on the successful functionalization of cotton fabrics with a water-stable metal–organic framework (MOF), UiO-66, under mild solvothermal conditions (80 °C) and its ability to adsorb and degrade water micropollutants. The functionalized cotton samples were characterized by X-ray diffraction (XRD), scanning electron microscopy (SEM), energy-dispersive X-ray spectroscopy (EDX), transmission electron microscopy (TEM), Fourier transform infrared spectroscopy (FTIR), and X-ray photoelectron spectroscopy (XPS). UiO-66 crystals grew in a uniform and conformal manner over the surface of the cotton fibers. The cotton fabrics functionalized with UiO-66 frameworks exhibited an enhanced uptake capacity for methylchlorophenoxypropionic acid (MCPP), a commonly used herbicide. The functionalized fabrics also showed photocatalytic activity, demonstrated by the degradation of acetaminophen, a common pharmaceutical compound, under simulated sunlight irradiation. These results indicate that UiO-66 can be supported on textile substrates for filtration and photocatalytic purposes and that these substrates can find applications in wastewater decontamination and micropollutant degradation.

## 1. Introduction

Metal–organic frameworks (MOFs) are highly tunable, porous materials with applications in gas adsorption [1,2,3,4,5,6], separation [7,8,9], catalysis [10,11,12,13], and filtration [14]. Zirconium-based MOFs in particular are of great interest because of their thermal stability [15] and their robustness over large ranges of pH [16]. UiO-66, a zirconium-based MOF, has excellent stability in water [17], making it an excellent candidate for the removal of micropollutants in aqueous systems [18,19,20]. However, the fact that these MOFs are synthesized almost exclusively in powder form may hinder their potential in applications requiring the use of large and mechanically stable, yet flexible surfaces. Textiles are mechanically stable and flexible substrates that can be manufactured in large volumes.

While the growth of MOFs on metal oxides has been amply reported, only few of these reports have discussed the coordination of the metal in the MOFs to fibers or textiles [21,22,23,24,25,26,27]. In one of these few reports, Zhao et al. [25] describe a method for the formation of MOF–nanofiber kebabs on Nylon-6 nanofibers using atomic layer deposition (ALD). ALD is a highly effective technique for precision coating, but because it requires the use of sophisticated equipment under controlled vacuum conditions, it remains expensive and rather difficult to scale up to large surfaces.

**Figure 1:**
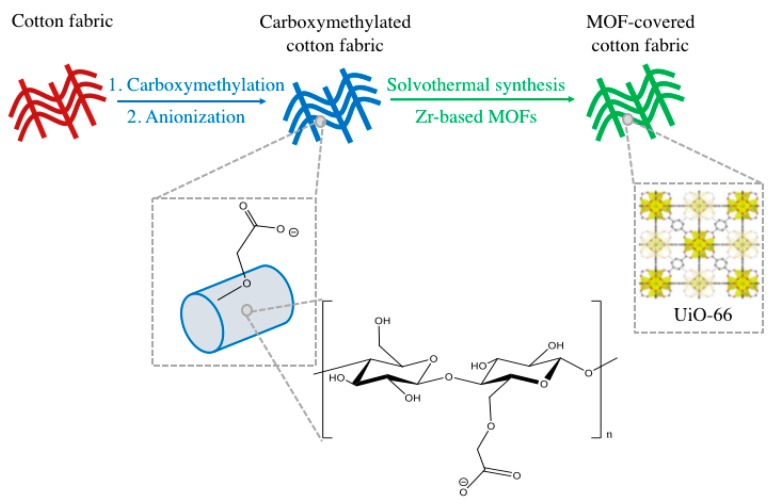
Schematic of the two-step synthesis pathway to grow UiO-66 on the surface of cotton fibers: The first step is the carboxymethylation of cellulose, and the second step is the low-temperature solvothermal synthesis of the metal-organic framework (MOF) on the surface of the carboxymethylated fibers.

Herein, we report on the growth of UiO-66 on cotton, using methods amenable to existing manufacturing processes used in the textile industry. Figure 1 shows the two-step synthesis pathway followed: The first step is the carboxymethylation of the cotton substrates [21], and the second step is the low-temperature solvothermal synthesis of the MOF on the surface of the carboxymethylated fibers.

Methylchlorophenoxypropionic acid (MCPP) is commonly used as a phenoxyacid herbicide, and it has been frequently detected in groundwater sources [28]. This contaminant can be partially removed by processes such as advanced oxidation and granular activated carbon filtration [29,30]. However, further improvements are needed because of issues related to the effectiveness, cost and environmental sustainability of these processes [31,32,33]. Seo et al. published promising results showing that the adsorption rate of MCPP onto UiO-66 (powder) was about 70 times faster than the adsorption rate of MCPP onto activated carbon. In the same report, it was shown that the adsorption capacity of UiO-66 was 7 times higher than that of activated carbon, particularly at low concentrations of MCPP [34].

In addition to herbicides, the presence of pharmaceutical products, such as acetaminophen (paracetamol), in natural waters, drinking water, and sewage, as well as in industrial and household waste streams has increased as a result of the growing consumption of antibiotics, anti-inflammatories and other readily available drugs. The long-term effects of exposure to pharmaceutical products in drinking water have not yet been fully determined, and the removal of these micropollutants from water remains a priority. In this manuscript, we evaluate the ability of cotton fabrics decorated with UiO-66 to absorb MCCP and to decompose acetaminophen.

## 2. Materials and Methods

All reagents were purchased from commercial sources and were used without further purification as follows: zirconium(IV) chloride (ZrCl_4_, ≥99.5%; Aldrich, St. Louis, MO, USA), terephthalic acid (98%; Alfa Aesar, Haverhill, MA, USA), *N*,*N*-dimethylformamide (DMF; Mallinckrodt Chemicals, Phillipsburg, NJ, USA), deionized (DI) water, sodium hydroxide (NaOH pellets; Macron), non-ionic surfactant Triton X-100 (0.08%; Electron Microscopy Sciences, Hatfield, PA, USA), isopropyl alcohol (Aldrich), acetic acid (Macron, glacial), sodium chloroacetate (98%; Aldrich), hydrochloric acid (HCl, 36.5–38%; J.T. Baker), methylchlorophenoxypropionic acid (Mecoprop, Santa Cruz Biotechnology, Dallas, TX, USA), and acetaminophen (Spectrum Chemical MFG Corp., New Brunswick, NJ, USA). Standardized cotton fabrics TIC-400 were obtained from Testfabrics, Inc. (West Pittston, PA, USA) and were cut into 2 × 2 cm^2^ squares using a laser cutter.

### 2.1. Scouring

A scouring solution was prepared by dissolving 5 g of NaOH, 1.5 g of Triton X and 0.75 g of acetic acid in 500 mL of DI water. The cotton swatches were immersed in the scouring solution at 100 °C following a procedure reported by Ozer et al. [35]. After scouring, the swatches were rinsed with DI water and hung to dry at room temperature.

### 2.2. Carboxymethylation

Carboxymethylation of the cotton swatches was performed by slightly modifying a method reported by Pushpamalar et al. [36]. The scoured cotton swatches were dipped in 100 mL of isopropyl alcohol; 10 mL of 30% (*w*/*v*) sodium hydroxide was added drop-wise and stirred for 1 h at room temperature. The solution was heated up to 45 °C, and 6.0 g of sodium monochloroacetate was added the reaction flask. After 3 h of vigorous stirring, the cotton swatches were cured at 85 °C for 30 min. The cotton swatches were washed with DI water and hung to dry at room temperature. Anionization of the substrates was achieved by dipping the modified cotton swatches in a solution of diluted acetic acid (0.2 mL in 100 mL of H_2_O) for 5 min, followed by washing the swatches with water and hanging at room temperature.

### 2.3. Growth of UiO-66 on the Surface of Cotton Fabrics

UiO-66 was prepared according to the procedure reported by Katz et al. [37]. The metal precursor solution was prepared by dissolving ZrCl_4_ (125 mg, 0.54 mmol) in a mixture of 5 mL of DMF and 1 mL of concentrated HCl and sonicating for 10 min. In a separate beaker, terephthalic acid (123 mg, 0.75 mmol) was dissolved in 10 mL of DMF and added to the metal precursor solution. Deprotonated carboxymethylated cotton swatches were dipped into the solution and left overnight at 80 °C in a tightly sealed container. The resulting fabric was washed in DMF (1 h) and H_2_O (1 h) and was dried at room temperature. UiO-66 in powder form was obtained under the same conditions as are described above but without the addition of the cotton swatches to the reaction vessel.

### 2.4. Characterization of the Samples

X-ray diffraction (XRD) experiments were performed on a Bruker D8 powder diffractometer with a step size of 0.04°. X-ray photoelectron spectroscopy (XPS) was performed using a Surface Science Instruments SSX-100 with an operating pressure of ~2 × 10^−9^ Torr. Monochromatic Al Kα X-rays (1486.6 eV) with a 1 mm diameter beam size were used. Photoelectrons were collected at a 55° emission angle. A hemispherical analyzer determined the electron kinetic energy using a pass energy of 150 V for wide/survey scans and of 50 V for high-resolution scans. A flood gun was used for charge neutralization.

Scanning electron microscopy (SEM) and energy-dispersive X-ray spectroscopy (EDX) were performed on a LEO 1550 FESEM (Keck SEM); the specimens were coated with a thin layer of Au-Pd or carbon for SEM and EDX. SEM imaging required an electric potential of 5 keV and an aperture of 30 µm, while EDX was performed using 7 keV and a 240 µm aperture. Transmission electron microscopy (TEM) was performed on a 120 kV field FEI T12 Spirit transmission electron microscope equipped with a LaB_6_ filament, single and double tilt holders, a SIS Megaview III CCD camera, and a STEM dark field and bright field detector. For the preparation of the TEM samples, the textile samples were immobilized in an epoxy resin mold and cured at 65 °C for 24 h. The immobilized sample was cut into 90 nm thick slides using an ultra-microtome and a diamond knife. Fourier transform infrared spectroscopy (FTIR) spectra were obtained with a Nicolet iS5 FTIR (Thermo Fisher Scientific) in ATR mode.

### 2.5. MCPP Uptake Experiments

Four cotton swatches functionalized with UiO-66 (0.2341 g) and four cotton swatches that underwent carboxymethylation (0.2349 g) were cut into small pieces using a laser cutter and were dried overnight in an oven at 100 °C. The swatches were immersed into aqueous solutions of MCPP (20 ppm, with a pH of 4.0 adjusted with 0.1 M HCl), which were prepared following the procedure reported by Seo et al. [34]. After 7 h and 24 h of vigorous stirring, the uptake of MCPP was determined using the UV absorbance at 279 nm.

### 2.6. Degradation of Acetaminophen

Acetaminophen (500 mg) was dissolved in 10 mL ethanol. Unmodified cotton samples (controls) and cotton samples functionalized with UiO-66 were dipped into the acetaminophen solution for 1 min. The wet samples were dried at 80 °C for 20 min and exposed for 10 min to a Xenon lamp to simulate solar conditions.

## 3. Results

### 3.1. Characterization of Cotton Fabrics Fucntionalized with UiO-66

#### 3.1.1. X-ray Diffraction

**Figure 2:**
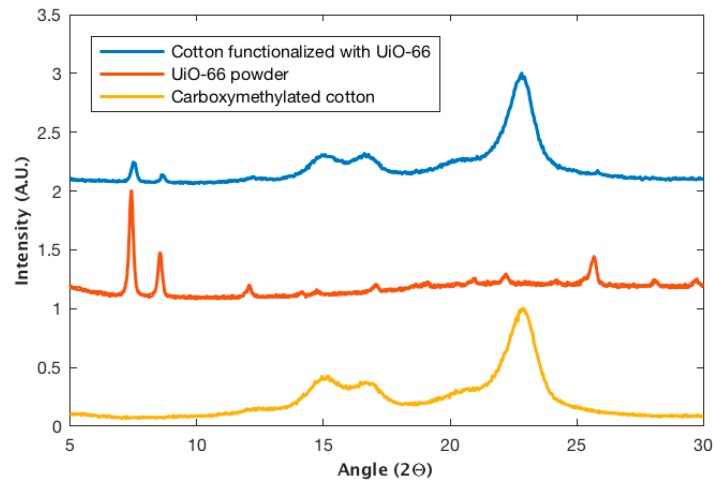
X-ray diffraction patterns for carboxymethylated cotton, UiO-66 powder, and cotton fabric functionalized with UiO-66.

The presence of the highly crystalline structure of the UiO-66 MOF was confirmed using XRD, as shown in Figure 2. The diffraction patterns of functionalized cotton samples (blue) and the pure UiO-66 (red) powder are shown in Figure 2 [15,38]. A clear superposition of the main diffraction peaks can be observed.

#### 3.1.2. X-ray Photoelectron Spectroscopy

XPS was used to determine the presence of zirconium, which is metal precursor for UiO-66.

**Figure 3:**
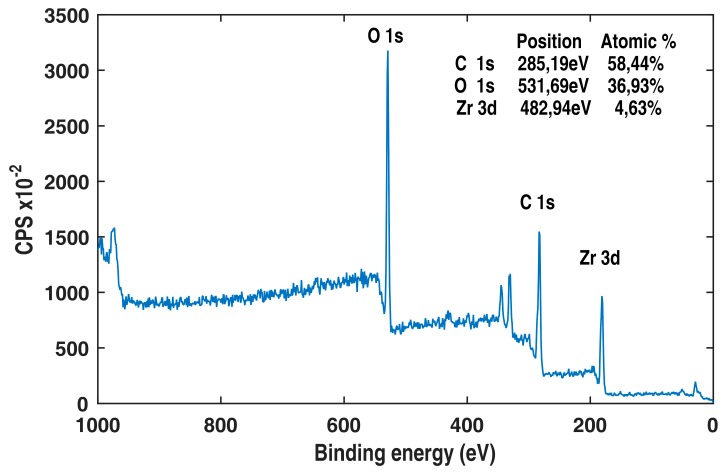
X-ray photoelectron spectroscopy (XPS) of a cotton fabric functionalized with UiO-66.

In Figure 3, the peaks at 330, 350 and 440 eV are assigned to zirconium’s 3p, 3p3/2 and 3s orbital electrons. The peak at 290 eV associated to carbon was used for calibration. This carbon peak can be assigned to both the ligand (terephthalic acid) and the substrate (cellulose). The 1s orbital of oxygen has a binding energy of 530 eV, and oxygen is also present in both the ligand and substrate.

As cotton samples are non-conductive, a buildup of positive charges on the surface of the sample can be formed immediately after the sample is exposed to X-rays. This charge buildup drags additional electrons from within the sample, hence distorting the spectrum and shifting down the binding energy values. A flood gun was used to neutralize this effect, and the spectrum was recalibrated using the C–C carbon bond at 285 eV. In addition to confirming the presence of Zr, the XPS spectra also indicated the absence of residual chlorine from the ZrCl_4_ and residual nitrogen that could have originated by the washing of the samples in DMF.

#### 3.1.3. Scanning Electron Microscopy

**Figure 4:**
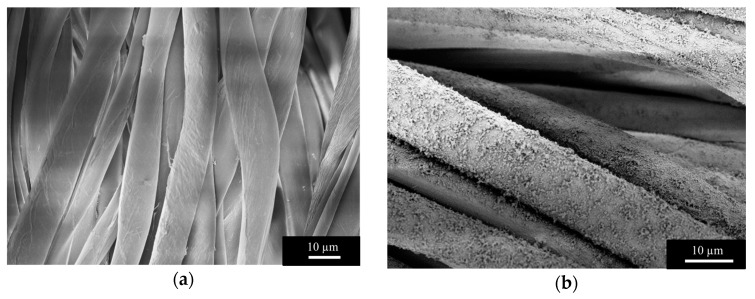

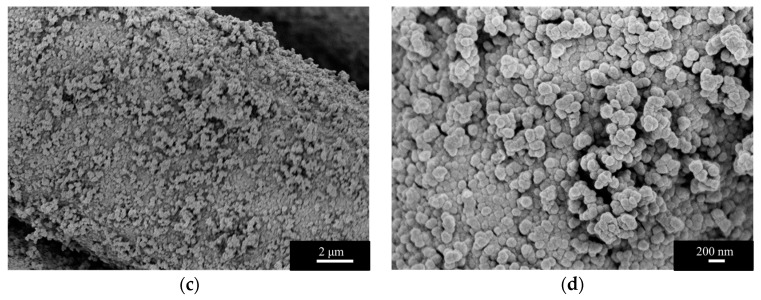
Scanning electron microscopy (SEM) images of (**a**) bare cotton fibers, and (**b**–**d**) cotton fibers functionalized with UiO-66 at different magnifications.

SEM imaging confirmed the uniformity of the UiO-66 functionalization on the surface of the cotton fabrics, as shown in Figure 4b,c. An SEM image of pristine cotton fibers is shown in Figure 4a for reference [38].

SEM imaging confirmed the presence of crystalline UiO-66. Figure 4d resembles the morphology previously reported for UiO-66 [17] and provides evidence of the homogeneity of the coating.

#### 3.1.4. Energy Dispersive X-ray Spectroscopy

**Figure 5:**
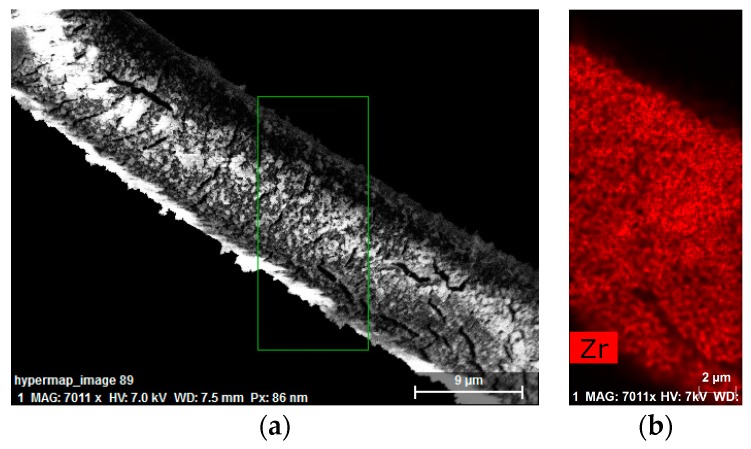
(**a**) Scanning electron microscopy (SEM) image of a cotton fiber functionalized with UiO-66 with the energy-dispersive X-ray spectroscopy (EDX) mapped region highlighted in green; (**b**) EDX zirconium map of the highlighted area shown in (**a**).

EDX mapping shows the atomic distribution over the surface of a sample. A higher aperture was used to ensure optimal characterization, but this also resulted in cracks in the sample, which are apparent in Figure 5a. Fortunately, once formed, these crevasses did not expand. A uniform distribution of zirconium onto the cotton fibers can be seen in Figure 5b, where the signal attributed to the zirconium channel is highlighted in red. Quantitative analysis of the resulting maps showed an average zirconium atomic percent content of 5% ± 0.7% on the surface of the fibers.

#### 3.1.5. Transmission Electron Microscopy

**Figure 6:**
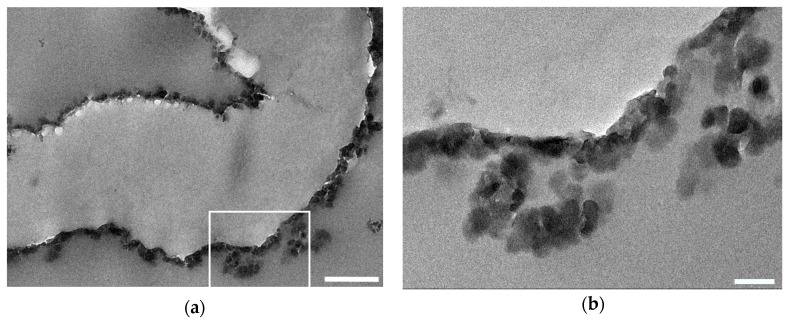
Transmission electron microscopy (TEM) image of a cotton fiber functionalized with UiO-66: (**a**) Cotton fiber cut along its fiber axis. The scale bar corresponds to 500 nm. (**b**) Magnification of the area delimited by the white square in (**a**). The scale bar corresponds to 100 nm.

TEM imaging allowed to assess the thickness of the UiO-66 coating on the cotton fibers. In Figure 6, the UiO-66 crystals, appearing darker than the cotton substrates because of the presence of Zr (Z = 40), formed a conformal coating over the irregular shape of the cotton fiber. These images were used to determine that the thickness of the coating was around 50 nm.

#### 3.1.6. Fourier Transform Infrared Spectroscopy

**Figure 7:**
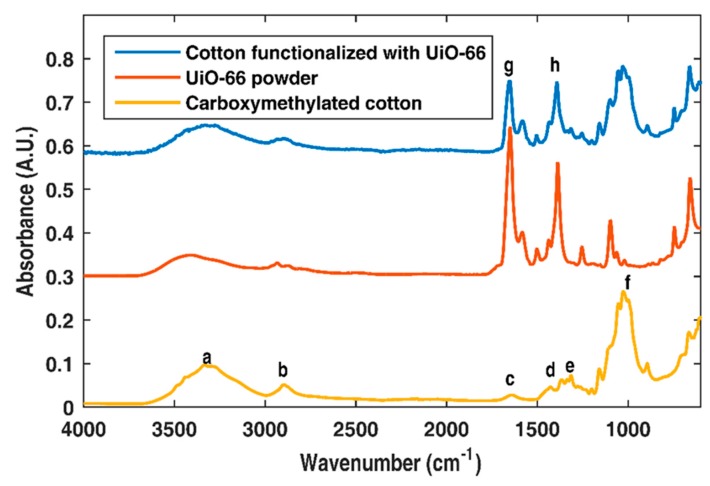
Fourier transform infrared spectroscopy (FTIR) spectra of bare cotton fabric, UiO-66 powder and UiO-66-functionalized cotton fabric.

The degradation of acetaminophen was followed using ATR-FTIR. The ATR-FTIR characterization of the UiO-66-functionalized fabrics is shown in Figure 7.

The labeled FTIR bands in Figure 7 correspond to (a) stretching of hydrogen-bonded O–H, (b) C–H stretching, (c) O–H bending of adsorbed water, (d) CH_2_ scissoring, (e) O–H bending (f) OCH–O–CH_2_ stretching, (g) asymmetric stretching of the carboxylate band (terephthalic ligand), and (h) carboxylate symmetrical stretching (terephthalic ligand). The powder spectrum and the carboxymethylated cellulose were in quantitative agreement with those previously reported [17,36,39].

### 3.2. Adsorption of MCPP

The maximum uptake of MCPP by dispersed UiO-66 powders was obtained after about 6 h according to Seo et al. [34]. In our case, the UiO-66 MOF was immobilized onto cotton fabrics. After immersion of the fabrics in the MCPP solution, an aliquot of the MCCP solution was taken after 7 h of vigorous stirring, and another aliquot was taken 24 h later. In both cases, the resulting UV absorption was the same. The calculated removal efficiency for the cotton fibers decorated with UiO-66 was 14.5%. As expected, the removal efficiency for the carboxymethylated cotton samples was 0% for uptake times as large as 24 h.

### 3.3. Photocatalytic Activity of the UiO-66-Functionalized Fabric

**Figure 8:**
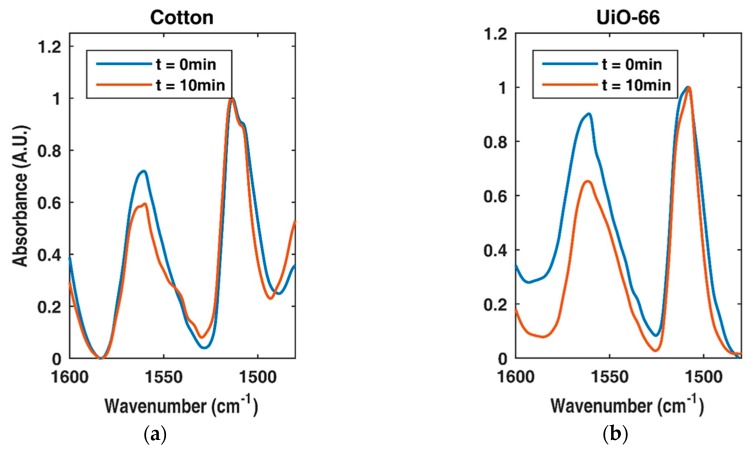
Acetaminophen adsorbed on (**a**) bare cotton fabric and (**b**) cotton fabrics functionalized with UiO-66. The Fourier transform infrared spectroscopy (FTIR) spectra in blue represents time zero and the spectra after 10 min of exposure to UV is shown in red.

The catalytic activity of the cotton fabrics functionalized with UiO-66 was assessed through degradation of acetaminophen. Figure 8 shows the absorbed and degraded acetaminophen after 10 min of UV exposure.

The degree of degradation of acetaminophen was evaluated by monitoring the 1560 cm^−1^ peak, which is ascribed to one of the N–H bending modes. The signal was normalized to the 1500 cm^−1^ peak attributed to one of the phenyl C–C stretching modes, a peak that should remain constant during photocatalytic degradation [40,41].

## 4. Discussion

XRD spectra of cotton samples functionalized with UiO-66 confirmed the crystalline formation of the MOF on the cotton fabric, as shown in Figure 2. Figure 2 also shows that the XRD pattern of the carboxymethylated cotton sample (yellow) exhibited only the characteristic peaks for cellulose (15°, 16° and 23°) [38]. The UiO-66 powder sample (red) matched the peaks reported in the literature [17]. The cotton sample functionalized with UiO-66 (blue) exhibited both the peaks assigned to cellulose and those assigned to UiO-66 in clear superposition, confirming the presence of the crystalline MOF onto the fabric.

XPS (Figure 3) confirmed that the crystalline formation was composed of zirconium, carbon and oxygen. Because of the fact that the substrate, cellulose, was also composed of carbon and oxygen, a quantitative analysis of the MOF content was not possible. The XPS spectra also showed no chlorine, confirming that the zirconium was stoichiometrically coordinated to the oxygen. Moreover, because of the absence of nitrogen in the spectrum, we also concluded that the solvent, DMF, was completely removed from the pores of the UIO-66 framework.

Images obtained via SEM (Figure 4) showed that the coating was uniform across the fabric, and this observation was supported by the EDX map reporting the distribution of zirconium (Figure 5b). Figure 4c,d show that the UiO-66 MOF completely covered the surface of the fiber, leaving no bare cotton in sight. EDX quantitative analysis indicated a zirconium content on the surface of 5% ± 0.7%.

TEM images (Figure 6) show that the thickness of the coating could be estimated to be around 50 nm and that the coating thickness appeared to be uniform along the fiber’s axis.

FTIR (Figure 7) spectra demonstrated the presence of carboxylate and phenyl bonds ascribed to the terephthalic ligands of UiO-66, and the spectra of the powder MOF and cellulose quantitatively agreed with the spectra reported in the literature [17,36]. Moreover, the spectrum of the cotton fabrics functionalized with UiO-66 showed all absorption peaks ascribed to both cellulose and UiO-66, further confirming the presence of this MOF onto the fabric. FTIR characterization was necessary for establishing a baseline for the photocatalytic experiment.

As illustrated by the EDX and XPS results, the amount of MOF immobilized onto the cotton fabric was very small. Therefore, a 14.5% uptake of MCPP is truly remarkable. In their study, Seo et al. reported a maximal adsorption capacity of 370 mg of MCPP/g UiO-66 (powder) [34]. In this study, we used 234.1 mg of a UiO-66-functionalized cotton containing about 5% zirconium on its surface. The mass of MCPP removed from the system was 3 mg, resulting in an adsorption capacity for UiO-66-functionalized cotton of 12.8 mg of MCPP/g functionalized fabric. As expected, the absorption capacity for the functionalized fabric was smaller than the value obtained for the powder sample. Advantages of the fabric sample include its easy recovery and reuse after desorption as well as its mechanical stability and manufacturability.

The catalytic activity of the cotton samples functionalized with UiO-66 MOFs was assessed in the degradation of acetaminophen. The degree of degradation was evaluated via FTIR by monitoring the 1560 cm^−1^ peak, which is ascribed to one of the N–H bending modes in acetaminophen. The signal was normalized to the 1500 cm^−1^ peak, which is ascribed to one of the phenyl C–C stretching modes. This peak should remain constant during the photocatalytic degradation [41]. Experiments were performed with the scoured cotton samples (control) and the cotton samples functionalized with UiO-66. In both experiments, the photocatalytic degradation of acetaminophen was noted (Figure 8). However, when the cotton sample functionalized with UiO-66 was used, the decrease in the N–H band was larger than in the experiments performed with the as-received samples. These results indicate that the presence of the UiO-66 increased the photocatalytic efficiency of the cotton by 63% after only 10 min of UV exposure.

## 5. Conclusions

Cotton fibers were successfully functionalized with UiO-66, a zirconium-based water-stable MOF. The presence of the crystalline UiO-66 was confirmed via XRD, and the presence of zirconium in the cotton-modified samples was confirmed by XPS and EDX. SEM and TEM imaging confirmed that the UiO-66 crystals uniformly grew on the surface of the cotton fibers and that the MOF layer was 50 nm thick. The reported functionalization did not alter the structure of the textile, hence allowing for the potential use of these materials in protective clothing. The cotton functionalized with UiO-66 exhibited an increased uptake of MCPP as well as enhanced UV degradation capabilities. These results indicate that UiO-66-functionalized textiles can be used as flexible mantles for the photocatalytic decomposition and adsorption of low-concentration pollutants in water streams.

## References

[1] Rosi N.L., Eckert J., Eddaoudi M., Vodak D.T., Kim J., O’Keefe M., Yaghi O.M. (2003). Hydrogen Storage in Microporous Metal-Organic Frameworks. Science.

[2] Férey G. (2008). Hybrid porous solids: Past, present, future. Chem. Soc. Rev..

[3] Vellingiri K., Kumar P., Deep A., Kim K.H. (2017). Metal-organic frameworks for the adsorption of gaseous toluene under ambient temperature and pressure. Chem. Eng. J..

[4] Cmarik G.E., Kim M., Cohen S.M., Walton K.S. (2012). Tuning the adsorption properties of UiO-66 via ligand functionalization. Langmuir.

[5] Sculley J., Yuan D., Zhou H.-C. (2011). The current status of hydrogen storage in metal-organic frameworks—Updated. Energy Environ. Sci..

[6] Makal T.A., Li J.-R., Lu W., Zhou H.-C. (2012). Methane storage in advanced porous materials. Chem. Soc. Rev..

[7] Fu Y.-Y., Yang C.-X., Yan X.-P., Ma Q., Feng Y.Q., Chevreau H., Serre C., Rodrigues A.E. (2013). Incorporation of metal–organic framework UiO-66 into porous polymer monoliths to enhance the liquid chromatographic separation of small molecules. Chem. Commun..

[8] Chang N., Yan X.-P. (2012). Exploring reverse shape selectivity and molecular sieving effect of metal-organic framework UiO-66 coated capillary column for gas chromatographic separation. J. Chromatogr. A.

[9] Vahidi M., Rashidi A.M., Tavasoli A. (2017). Preparation of piperazine-grafted amine-functionalized UiO-66 metal organic framework and its application for CO_2_ over CH_4_ separation. J. Iran. Chem. Soc..

[10] Timofeeva M.N., Panchenko V.N., Jun J.W., Hasan Z., Matrosova M.M., Jhung S.H. (2014). Effects of linker substitution on catalytic properties of porous zirconium terephthalate UiO-66 in acetalization of benzaldehyde with methanol. Appl. Catal. A Gen..

[11] Liu Y., Howarth A.J., Hupp J.T., Farha O.K. (2015). Selective Photooxidation of a Mustard-Gas Simulant Catalyzed by a Porphyrinic Metal-Organic Framework. Angew. Chem. Int. Ed..

[12] Cirujano F.G., Luz I., Soukri M., van Goethem C., Vankelecom I., Lail M., de Vos D.E. (2017). Boosting catalytic performance of MOFs for steroid transformations by confinement within mesoporous scaffolds. Angew. Chem. Int. Ed..

[13] Katz M.J., Mondloch J.E., Totten R.K., Park J.K., Nguyen S.T., Farha O.K., Hupp J.T. (2014). Simple and compelling biomimetic metal-organic framework catalyst for the degradation of nerve agent simulants. Angew. Chem. Int. Ed..

[14] Barea E., Montoro C., Navarro J.A.R. (2014). Toxic gas removal—Metal-organic frameworks for the capture and degradation of toxic gases and vapours. Chem. Soc. Rev..

[15] Kandiah M., Nilsen M.H., Usseglio S., Jakobsen S., Olsbye U., Tilset M., Larabi C., Quadrelli E.A., Bonino F., Lillerud K.P. (2010). Synthesis and Stability of Tagged UiO-66 Zr-MOFs. Chem. Mater..

[16] Jiang H.-L., Feng D., Wang K., Gu Z.-Y., Wei Z., Chen Y.-P., Zhou H.-C. (2013). An Exceptionally Stable, Porphyrinic Zr Metal–Organic Framework Exhibiting pH-Dependent Fluorescence. J. Am. Chem. Soc..

[17] Cavka J.H., Jakobsen S., Olsbye U., Guillou N., Lamberti C., Bordiga S., Lillerud K.P. (2008). A New Zirconium Inorganic Building Brick Forming Metal Organic Frameworks with Exceptional Stability. J. Am. Chem. Soc..

[18] Chen C., Chen D., Xie S., Quan H., Luo X., Guo L. (2017). Adsorption Behaviors of Organic Micropollutants on Zirconium Metal-Organic Framework UiO-66: Analysis of Surface Interactions. ACS Appl. Mater. Interfaces.

[19] Hasan Z., Jhung H. (2015). Removal of hazardous organics from water using metal-organic frameworks (MOFs): Plausible mechanisms for selective adsorptions. J. Hazard. Mater..

[20] Shang H.-B., Yang C.-X., Yan X.-P. (2014). Metal–organic framework UiO-66 coated stainless steel fiber for solid-phase microextraction of phenols in water samples. J. Chromatogr. A.

[21] Da Silva Pinto M., Sierra-Avila C.A., Hinestroza J.P. (2012). In situ synthesis of a Cu-BTC metal-organic framework (MOF 199) onto cellulosic fibrous substrates: Cotton. Cellulose.

[22] Yu M., Li W., Wang Z., Zhang B., Ma H., Li L., Li J. (2016). Covalent immobilization of metal-organic frameworks onto the surface of nylon—A new approach to the functionalization and coloration of textiles. Sci. Rep..

[23] Bunge M.A., Ruckart K.N., Leavesley S., Peterson G.W., Nguyen N., West K.N., Glover T.G. (2015). Modification of Fibers with Nanostructures Using Reactive Dye Chemistry. Ind. Eng. Chem. Res..

[24] Lemaire P.C., Zhao J., Williams P.S., Walls H.J., Shepherd S.D., Losego M.D., Peterson G.W., Parsons G.N. (2016). Copper Benzenetricarboxylate Metal–Organic Framework Nucleation Mechanisms on Metal Oxide Powders and Thin Films formed by Atomic Layer Deposition. ACS Appl. Mater. Interfaces.

[25] Zhao J., Lee D.T., Yaga R.W., Hall M.G., Barton H.F., Woodward I.R., Oldham C.J., Walls H.J., Peterson G.W., Parsons G.N. (2016). Ultra-Fast Degradation of Chemical Warfare Agents Using MOF-Nanofiber Kebabs. Angew. Chem. Int. Ed..

[26] Lu A.X., McEntee M., Browe M.A., Hall M., DeCoste J.B., Peterson G.W. (2017). MOFabric: Electrospun Nanofiber Mats from PVDF/UiO-66-NH_2_ for Chemical Protection and Decontamination. ACS Appl. Mater. Interfaces.

[27] Lee D.T., Zhao J., Peterson G.W., Parsons G.N. (2017). A Catalytic ‘MOF-Cloth’ Formed via Directed Supramolecular Assembly of UiO-66-NH_2_ Crystals on ALD-coated Textiles for Rapid Degradation of Chemical Warfare Agent Simulants. Chem. Mater..

[28] Ignatowicz K. (2009). Selection of sorbent for removing pesticides during water treatment. J. Hazard. Mater..

[29] Suty H., de Traversay C., Cost M. (2004). Applications of advanced oxidation processes: Present and future. Water Sci. Technol..

[30] SHeijman G.J., Siegers W., Sterk R., Hopman R. (2002). Prediction of breakthrough of pesticides in GAC-filters and breakthrough of colour in ion-exchange-filters. Water Sci. Technol. Water Supply.

[31] Godskesen B., Zambrano K.C., Trautner A., Johansen N.B., Thiesson L., Andersen L., Clauson-Kaas J., Neidel T.L., Rygaard M., Kløverpris N.H. (2011). Life cycle assessment of three water systems in Copenhagen—A management tool of the future. Water Sci. Technol..

[32] Hedegaard M.J., Arvin E., Corfitzen C.B., Albrechtsen H.J. (2014). Mecoprop (MCPP) removal in full-scale rapid sand filters at a groundwater-based waterworks. Sci. Total Environ..

[33] Petrović M., Gonzalez S., Barceló D. (2003). Analysis and removal of emerging contaminants in wastewater and drinking water. TrAC—Trends Anal. Chem..

[34] Seo Y.S., Khan N.A., Jhung S.H. (2015). Adsorptive removal of methylchlorophenoxypropionic acid from water with a metal-organic framework. Chem. Eng. J..

[35] Athauda T.J., Hari P., Ozer R.R. (2013). Tuning Physical and Optical Properties of ZnO Nanowire Arrays Grown on Cotton Fibers. ACS Appl. Mater. Interfaces.

[36] Pushpamalar V., Langford S.J., Ahmad M., Lim Y.Y. (2006). Optimization of reaction conditions for preparing carboxymethyl cellulose from sago waste. Carbohydr. Polym..

[37] Katz M.J., Brown Z.J., Colón Y.J., Siu P.W., Scheidt K.A., Snurr R.Q., Hupp J.T., Farha O.K. (2013). A facile synthesis of UiO-66, UiO-67 and their derivatives. Chem. Commun..

[38] Zhao H., Kwak J., Conradzhang Z., Brown H., Arey B., Holladay J. (2007). Studying cellulose fiber structure by SEM, XRD, NMR and acid hydrolysis. Carbohydr. Polym..

[39] Biswal D.R., Singh R.P. (2004). Characterisation of carboxymethyl cellulose and polyacrylamide graft copolymer. Carbohydr. Polym..

[40] Kandiah M., Usseglio S., Svelle S., Olsbye U., Lillerud K.P., Tilset M. (2010). Post-synthetic modification of the metal–organic framework compound UiO-66. J. Mater. Chem..

[41] Amado A.M., Azevedo C., Ribeiro-Claro P.J.A. (2017). Conformational and vibrational reassessment of solid paracetamol. Spectrochim. Acta.

